# Motivational profiles of kindergarten teachers in minority areas of China and their association with outcomes

**DOI:** 10.3389/fpsyg.2022.1023073

**Published:** 2022-10-25

**Authors:** Dasheng Shi, Mengmeng Zhang, Ye Chen, Ruining Jin, Xiantong Yang

**Affiliations:** ^1^School of Education, Minzu University of China, Beijing, China; ^2^Department of Preschool Education, Teachers’ College, Beijing Union University, Beijing, China; ^3^Civil, Commercial and Economic Law School, China University of Political Science and Law, Beijing, China; ^4^Faculty of Psychology, Beijing Normal University, Beijing, China

**Keywords:** kindergarten teacher motivation, work engagement, workplace wellbeing, retention intention, minority

## Abstract

Academic discourses regarding teacher motivation have been on-going for decades for those who teach in ethnic minority areas. Yet research findings failed to provide a consistent conclusion regarding if kindergarten teachers’ motivation pattern would vary based on a case-to-case scenario. Therefore, further studies are needed to probe the motivation patterns among this population. The study firstly examined kindergarten teachers’ motivational profiles based on Expectancy Value Theory (EVT), and then examined how teachers’ motivation related to outcome variables (work engagement, workplace wellbeing, and retention intention). Participants included 1,199 kindergarten teachers from ethnic minority areas in China. Latent profile analysis identified three motivation profiles for teachers: low value-high cost (profile 1), moderate all (profile 2), and high value-low cost (profile 3). Teacher with different motivation profiles had significant differences in work engagement, workplace wellbeing, and retention intention. In addition, chain mediation analysis revealed that work engagement and workplace wellbeing mediated the relationship between motivation profiles and retention intention. The implications of the findings for study are discussed.

## Introduction

Teacher motivation in Chinese ethnic minority areas has been a focal point during domestic and international academic discourse (Wu et al., 2020; Long, 2021; Long and Yuan, 2021). Motivation is a critical component of teacher professionalism and a factor in teacher retention (Alexander et al., 2020). Previous research have substantiated the correlation between reduced motivation and lowered morale (Wang et al., 2019), which in turn would lower the overall education quality (Long and Yuan, 2021). Therefore, it is critical to investigate kindergarten teacher work motivation in ethnic minority areas to avoid the chain effects caused by insufficient motivation.

However, previous investigations have indicated that teachers’ work motivation had different impacts on their behaviors (Abós et al., 2018). A possible reason for that is that most studies on teacher motivation traditionally used a variable centered method, in which the influence of specific teacher motivation of teacher behavior has typically been explored discretely (e.g., Li and Gong, 2020; Hong et al., 2021). Such a methodology usually failed to take the possibility of unobservable subgroups into consideration and therefore provided a limited interpretation to the data. As a result, a thorough investigation that relies on the interaction of multiple motivating characteristics is imperative to an accurate apprehension of the overall phenomena of kindergarten teacher motivation, because motivational features do not operate independently upon one another; rather, they may influence teacher behavior in a multiplicative way (Wigfield et al., 2006). For instance, teachers teach not only because they need to obtain financial benefits and other welfare, but also because they find teaching enjoyable as such (Guo and Sun, 2018; Rad et al., 2022c). Therefore, kindergarten teachers’ motivation profiles may vary based on various motivational factors.

This study, under the framework of Expectancy Value Theory (EVT), uses a person-centered methodology to explore the motivation profiles of kindergarten teachers in ethnic minority areas in China. Person-centered approaches categorizes individuals into common profiles with similar motivational patterns (Thommen et al., 2021). This is especially accommodating when numerous variables are being studied simultaneously, while little is known about how they interact. Overall, the aims of this study were to (a) identify subgroups with similar motivational profiles using latent profile analysis, (b) explore the effect of motivation profiles on psychological outcomes, and (c) investigate the psychological mechanism underlying the association between motivation profiles and work engagement, workplace wellbeing, and retention intention.

### Teachers’ motivation from the expectancy value theory

Seen as differentiated and multi-dimensional, motivation is considered to be a combination of internal features and processes that would provide rationales behind individual’s behaviors (Pintrich, 2003). Different motivational theories have been used to the teaching profession by some scholars in order to examine the complexity of motivation (Thommen et al., 2021), including EVT- one of the most extensively utilized theory to describe individual motivational processes in an educational context (Osman and Warner, 2020). Action-driving motivation, as assumed by EVT, has two key components: expectancy for success and task values. Expectancy refers to an individual’s belief in his ability to successfully perform a task; Task values are subjective assessments of task priority, through which individuals could distinguish among attainment value, intrinsic value, utility value, and cost (Eccles and Wigfield, 1995, 2002). Attainment value is understood as the importance to the individual of finishing a task. Intrinsic value refers to the pleasure that an individual feel in completing a task. When appreciating utility value, one would usually gain a sense of success should the completion of a given task is in alignment with the fulfillment of one’s personal plan or goal. Finally, cost refers to the negative aspects of an individual undertaking a task, such as individual anxiety for the devotion required by the task or the negative outcomes of the tasks (Eccles and Wigfield, 2002). In summary, these components of expectancy and values converge to guide individuals to make behavioral decisions (Nagengast et al., 2013; Osman and Warner, 2020).

The EVT framework facilitates the examination of multiple motivational characteristics, as motivation for a particular behavior is an outcome of the combination of individual expectations and task values. For instance, some teachers may have high attainment value and consider teaching to be extremely rewarding (Collie and Martin, 2017), whereas others may have low utility value but maintain a high self-efficacy (Li, 2018). The interaction between the values and expectancy components explains why teachers are particularly motivated when both components score strongly (Thommen et al., 2021). More precisely, one component of motivation may partially compensate for another, reducing the negative impact on overall motivation (Trautwein et al., 2012). Thus, by utilizing EVT, the purpose of our study is to gain a thorough understanding of the key features of teacher motivation, which not only provides compelling evidence for a person-centered approach but may also have significant implications for teacher professional development and educational quality improvement.

### Teachers’ motivation: Toward a person-centered approach

Teachers’ motivations may vary across various instructional domains because of the unobservable subgroups and unique motivational subcomponents. Previous studies mostly focused on teacher motivation from a variable-centered perspective (Durksen et al., 2017; Ahn et al., 2021). This approach can be used to investigate the unique associations between two or more independent motivation variables and outcomes (e.g., engagement, wellbeing; Parker and Martin, 2009), but it requires that all samples belong to the similar population and have the same parameter estimates. This ignores the potential of unobservable subgroups and reduces the study validity due to incorrect findings interpretation. Therefore, a person-centered approach is needed to identify naturally co-existing subgroups of teachers who display a variety of motivational features.

Such an approach was used in previous research regarding teacher work motivations. For example, Wang et al. (2019) discovered four latent motivation profiles among Chinese special-post teachers: high intrinsic-high extrinsic motivation, high intrinsic-low extrinsic motivation, low intrinsic-high extrinsic motivation, and a balanced motivation. Thommen et al. (2021) investigated mathematics teachers’ work motivation and identified complex configuration of profiles: low performance goal oriented, high performance goal oriented, high performance-avoidance goal oriented. These studies indicated that teachers engage in their teaching for a variety of reasons. When it comes to teachers in China, due to the effects of job content, cultural variety, and geography, there may be differences in the motivations of kindergarten teachers in China’s ethnic minority areas and other groups (Cai and Yuan, 2018; Xia, 2020). Therefore, more study is needed to determine the various motivation profiles of kindergarten teachers in ethnic minority areas. Accordingly, we aimed to investigate profiles of kindergarten teacher motivation applying person-centered method.

### The possible association between teacher motivation profiles and outcomes and its underlying mechanism

Various motivation profiles would impact individual teachers based on a case-by-case scenario and therefore lead to different outcomes. From a social cognitive perspective, teachers would experience favorable outcomes (e.g., work engagement, workplace wellbeing and retention intention) when they possess a strong motivation to perform required tasks (Anthony and Ord, 2008). However, empirical data is insufficient for researchers to draw conclusions regarding the impacts of the identified motivation profiles of kindergarten teachers on the outcome variables. As teachers are expected to have different motivations across various domains (Gorozidis and Papaioannou, 2014), it is vital to investigate the impacts of profiles of motivation to provide a more comprehensive perspective to illustrate the effect mechanism of identified motivation profiles of kindergarten teachers on outcomes. Previous studies have shown that highly motivated teachers would generally hold greater self-esteem, which in turn, would lower their turnover rate (Torquati et al., 2007). Nonetheless, the influence of motivation profiles on engagement and workplace wellbeing has not been fully scrutinized, as the individual psychological performance influenced by motivation profile differs (Grant, 2008). Thus, it is necessary to clarify how the link between motivation profile and retention intention is affected by engagement and workplace wellbeing.

Work motivation has been highlighted as an important variable influencing teacher retention. Retention intention is individual’s perception of the likelihood of remaining in the same institution (Price and Mueller, 1981). It has been corroborated that teachers who were highly motivated at work were more likely to remain with the organization (Zhang et al., 2019). Another study with pre-service teachers demonstrated that the teaching intentions of teacher subgroups were more or less associated with different motivation profiles (Richardson and Watt, 2006). Thus, we hypothesize that teachers with varying motivation profiles will have varying influences on their retention intention.

Teachers’ motivation profiles may predict retention through teachers’ work engagement. Work engagement refers to a favorable attitude toward work highlighted by vigor, dedication, and absorption (Schaufeli et al., 2006). Based on Conservation of Resources Theory, motivation, as the processing resources, enables the accumulation of extra resources (Hobfoll, 2002). Work engagement as an additional accumulated resource has been found in job-related studies (Valero and Hirschi, 2016). For example, teachers’ autonomous motivation (defined as intrinsic) was found to be a positive predictor of work engagement by Li et al. (2015). Jansen in de Wal et al. (2014) examined the association between motivation profiles of secondary school teachers and their engagement, and found that “extremely autonomous” profile could most positively predict teacher engagement. Furthermore, regarding the link between work engagement and retention intention, earlier study revealed that engagement could exert potential effects on retention intention. One meta-analytical finding indicated a negative association between work engagement and attrition, indicating that work engagement might positively predict teacher’s retention intention (Halbesleben, 2010). According to the relevant literature, our present study speculated that work engagement possibly presents as a mediating link between motivation profiles and retention.

Moreover, motivation profiles may predict retention intention through teachers’ workplace wellbeing. Workplace wellbeing refers to individuals’ cognition of satisfaction at work, as well as their emotional and psychological experiences and health state at work (Zheng et al., 2015). According to the person-organization fit theory (Kristof, 1996), when employees’ motivations are highly aligned with those of their job, they are more likely to respond positively to organizational practices. Such an alignment would lead to an increase in optimism and positive attitudes at work, indicating job wellbeing (Fu et al., 2020). Howard et al.’s (2016) study also indicated that individuals with highly motivated profiles demonstrated improved work performance and higher wellbeing. Furthermore, research suggests that workplace wellbeing could exert potential effects on retention intention. According to Sears et al. (2013)’s model of wellbeing improvement and employer outcomes, the deterioration of work-related wellbeing was expected to cause employees’ psychological withdrawal from work, hampered performance, and ultimately in resignation (Sears et al., 2013; Bardach et al., 2022). Thus, this study hypothesizes that workplace wellbeing would mediate the association between motivation profiles and retention intention.

There may be a link between work engagement and workplace wellbeing. Work engagement is an essential strategy to attain self-actualization in the workplace through effort (Yang et al., 2019), whereas wellbeing is viewed as the consequence of self-actualization (Rothmann, 2008). Yang et al. (2019) indicated that employees with had higher work engagement perceived greater levels of employee wellbeing. Thus, employees’ work engagement has a favorable effect on their wellbeing (De-la-Calle-Durán and Rodríguez-Sánchez, 2021).

Work engagement and workplace wellbeing may jointly mediate the link between motivation profiles and retention intention. Job Demands-Resources model (Bakker et al., 2004) illustrated how engagement and workplace wellbeing would joint force to exert an impact on the correlation between motivation profiles and retention intention. Namely, greater wellbeing at work is determined by work resources via work engagement. Related studies showed that resources (such as self-efficacy as an aspect of motivation) are significantly associated with work engagement and wellbeing (Lee, 2019; Han et al., 2020). Similar studies also indicated that teacher with attainment value have greater positive work engagement and wellbeing. Furthermore, work engagement and wellbeing were predictor variables of retention (Brunetto et al., 2014; Fu et al., 2020). Taken together, it is reasonable to hypothesize that work motivation profiles could indirectly predict the retention intention of kindergarten teachers via the chain mediating roles of work engagement and workplace wellbeing.

To conclude, our third aim is to investigate the impact of motivation profiles on outcome variables. On this premise, exploring the process mechanism of motivation profiles impacting retention intention is our fourth research goal.

### Current study

Several gaps in existing studies on teacher motivation characteristics were discovered throughout our review of the literature. First, previous research in teacher groups found inconsistent motivational characteristics (Durksen et al., 2017; Ahn et al., 2021). Unique profiles may exist in the kindergarten teachers. Consequently, more investigations are required to confirm the profile characteristics of kindergarten teachers in ethnic minority areas in China. Second, empirical research on the career motivations of kindergarten teacher in ethnic minority areas in China remains limited. Furthermore, it is necessary to clarify the outcomes of differences in kindergarten teachers’ motivation profiles, and the complex mechanism between kindergarten teacher motivation profiles and retention intention. Responding to these gaps, the current study’s aims were quadruple: First (RQ 1), Given the paucity of person-centered research, latent profile analysis (LPA) is used to identify kindergarten teachers’ motivation profiles. Second (RQ 2), to extend previous study indicating that kindergarten teachers’ motivation profiles may affect their subjective experience of long-term teaching, we intended to evaluate if the motivation profile of kindergarten teachers is associated with their work engagement, workplace wellbeing, and retention intention. Lastly (RQ 3), to elaborate on the process by which the identified motivation profiles of kindergarten teachers influence the outcome variables, we attempted to illustrate the internal mechanism between motivation profiles and outcome variables. Through the above description, the following four research questions are investigated:

RQ 1: What motivation profiles appear among a sample of kindergarten teachers in ethnic minority regions in China?
RQ 2: Do teacher motivation profiles differ in different outcome variables (work engagement, workplace wellbeing, and retention intention)?
RQ 3: How do motivation profiles relate to work engagement, workplace wellbeing, and retention intention?

**FIGURE 1:**
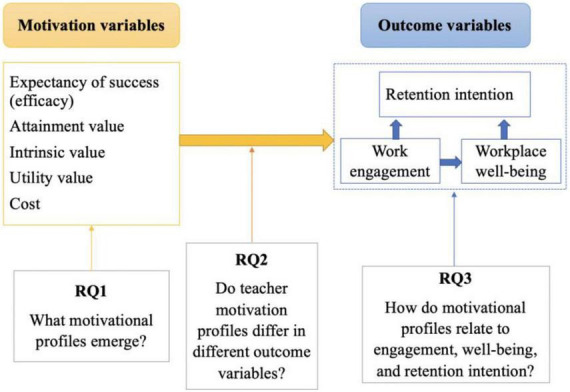
Theoretical model.

Figure 1 illustrates the theoretical model that guides current research questions. To respond to RQ1, a person-centered strategy is used to search for evidence of relatively homogeneous subgroups within the overall samples based on EVT and prior literature (Eccles and Wigfield, 2002; Wang et al., 2019). We hypothesized that the identified motivation profiles would reflect disparities in motivation levels among kindergarten teachers in China’s ethnic minority areas (high, medium, and low).

Regarding RQ2, we proposed that more favorable motivation profiles would be positively correlated with more positive work engagement, workplace wellbeing, and retention intention.

Concerning RQ3, we were interested in the mechanism underlying the association between distinct motivation profiles and retention intention. Based on previous theories (e.g., conservation of resources theory, etc.) and studies, we expected that a favorable motivation profile would enhance kindergarten teacher’s retention intention through a greater work engagement and workplace wellbeing. In contrast, we anticipated that the negative motivation profile would diminish kindergarten teacher’s retention intention through a lower work engagement and worsened workplace wellbeing.

## Materials and methods

### Participants and procedures

A total of 3,378 adolescents participated (Me = 15.60, SD = 1.51) in this study, include ageing 1,513 (44.70%) boys, 1,820 (54.00%) girls, and 45 participants (1.30%) with missing gender information; among them were 1,237 middle school students (seventh and eighth graders), 2,109 high school students (tenth and eleventh graders), and two participants with missing grade information. Participants and their parents received informed consent information from the school and were told they could leave the study whenever they desired. They completed the questionnaire in the classroom, and all measurements and procedures were approved by the Institutional Review Board (IRB) of the authors’ institution.

Convenience sampling was used to obtain kindergarten teachers’ data in ethnic minority areas of China in 2021. Teachers working in 32 kindergartens (such as Ningxia Hui Autonomous Region, Tibetan Autonomous Prefecture, etc.) were sampled. All respondents were notified that their participation in this research was entirely voluntary and the study was for academic purposes. A total of 1,199 valid questionnaires were received. Among the participants, 1,159 identified themselves as female (96.7%), and 40 identified themselves as male (3.30%). Regarding educational level, 283 participants reported to be post-secondary graduates/high school graduates, and 911 (76%) self-identified as undergraduates and 5 (0.4%) claimed to be postgraduates. Regarding teaching experience, 878 teachers (73.2%) had less than 2 years of teaching experience, while 321 teachers (26.8%) had more than 2 years of teaching experience. 768 (65%) were Han, 402 (33.5%) were Hui, and the rest of the samples were from other ethnic groups (such as Bai, Tibetan, Dai, etc.). The institution of the first author’s Institutional Review Board (IRB) authorized the research procedure.

## Measures

### Teaching motivation

The EVT created by Eccles and Wigfield (2002) was used to measure kindergarten teachers’ teaching motivation in ethnic minority areas in China. The questionnaire included five subcomponents: Expectancy of success (self-efficacy, e.g., “I’m fit to be a kindergarten teacher.”), attainment value (e.g., “Being a kindergarten teacher allows me to contribute to society.”), intrinsic value (e.g., “I adore working with kids.”), utility value (e.g., “I got a decent salary for local kindergarten.”), and cost (e.g., “Kindergarten teachers have less competitive pressure.”). A five-point Likert scoring were applied, with 1 indicating “*completely disagree*” and five indicating “*completely agree.*” Cronbach’s alphas for the motivation components ranged from 0.795 to 0.892 in this study. CFA showed a good fit, χ*^2^/df* = 3.29, *p* < 0.001, RMSEA = 0.044, CFI = 0.989, TLI = 0.982, SRMR = 0.026.

### Working engagement

The Utrecht Work Engagement Scale (Schaufeli et al., 2006) was used to assess work engagement, it includes three subdimensions, namely vigor (two items, e.g., “I feel energetic at work.”), dedication (three items, e.g., “I am passionate about my work.”), and absorption (two items, e.g., “I was drowned in my work.”). A five-point Likert scoring were applied, with one indicating “*completely disagree*” and five indicating “*completely agree*.” Cronbach’s alpha for this scale was 0.951. CFA showed a good fit, χ*^2^/df* = 3.6, *p* < 0.001, RMSEA = 0.047, CFI = 0.996, TLI = 0.994, SRMR = 0.008.

### Workplace wellbeing

The workplace wellbeing scale was established by Zheng et al. (2015) to assess kindergarten teachers’ workplace wellbeing (six items, e.g., “In general, I’m content with my current employment.”). A five-point Likert scoring were applied, with 1 indicating “*completely disagree*” and five indicating “*completely agree*.” In the current investigation, Cronbach’s alpha was 0.963 for this scale. CFA showed a good fit, χ*^2^/df* = 8.3, *p* < 0.001, RMSEA = 0.078, CFI = 0.992, TLI = 0.987, SRMR = 0.009.

### Retention intention

The retention intention of kindergarten teachers was measured using the retention scale developed by Guo and Sun (2018), which consists of four items (e.g., “I intend to continue to teach in kindergarten”). A five-point Likert scoring were applied, with one indicating “*completely disagree*” and five indicating “*completely agree.*” The Cronbach’s alpha in current research was 0.910. CFA revealed a good fit, χ*^2^/df* = 2.9, *p* < 0.001, RMSEA = 0.040, CFI = 0.998, TLI = 0.995, SRMR = 0.009.

## Data analysis

For preliminary data processing, descriptive statistics, correlation analysis, and multinomial logistic regression, SPSS 23.0 was utilized. Mplus 7.4 was used to run CFA, LPA, and mediation analysis using the robust maximum likelihood estimation (MLR) (Muthén and Muthén, 1998). To evaluate model fit, the following fit indices were used: The Chi squared goodness of fit test (χ^2^), the comparative fit index (CFI), the Tucker-Lewis index (TLI), the root means square error of approximation (RMSEA), and the standardized root mean square residual (SRMR). The cutoff criteria suggested by Hu and Bentler (1999), CFI ≥ 0.90, TLI ≥ 0.90, RMSEA < 0.08, SRMR < 0.1, were used to evaluate the data fit (Chen, 2007).

To answer the RQ 1, we conducted LPA to identify teachers’ work motivation profiles. First, one to five class models were examined after the probability profiles of teacher motivation were run with an unconditional LPA. Second, a number of statistical measures, including the Akaike information criterion (AIC), Bayesian information criterion (BIC), sample-size adjusted BIC (aBIC), Vuong-Lo-Mendell-Rubin-likelihood ratio test (VLMR), Lo-Mendell Rubin adjusted LRT test (LMR-A), Bootstrap likelihood ratio test (BLRT), and entropy, were used to identify the best solution. Lower AIC, BIC, and aBIC generally indicated better fit of the model. Entropy, which ranges from 0 to 1, described the precision of the categorization, with a larger number suggesting more accuracy (McMullen et al., 2018). The *p*-values of BLRT and VLMRT were significant, showing that k profile model suited the data better than the K-1 profile model (Nylund et al., 2007).

To address the RQ2, we investigated how motivation profiles influence outcome variables (work engagement, workplace wellbeing, and retention intention). Variance analysis was used to investigate differences in outcome variables between latent profiles.

Regarding the RQ 3, we conducted mediation analysis (Hayes, 2013) to determine whether the effect of teaching motivation profiles on retention intention passes through work engagement and workplace wellbeing after controlling for age and gender. The significance level of the indirect effect was assessed using bootstrapping approaches. A bias-corrected bootstrap confidence interval of 95% excluding zero implied that the indirect influence is significant.

## Results

### Preliminary analyses

Latent variable correlations were displayed in Table 1. Modified CFA showed a suitable fit, χ*^2^/df* = 10.17, *p* < 0.001, RMSEA = 0.087, CFI = 0.934, TLI = 0.899, SRMR = 0.077. All indicators were found to be significantly linked with one another. The average scores of intrinsic value, attainment value, utility value, self-efficacy, and cost had modest correlations, indicating that LPA was appropriate.

**TABLE 1:** Correlations of latent variables.

	1	2	3	4	5	6	7	8
1. Intrinsic value	1							
2. Attainment value	–0.436***	1						
3. Utility value	0.479***	–0.212**	1					
4. Self-efficacy	–0.249**	0.531***	–0.209**	1				
5. Cost	–0.315**	0.180**	–0.623***	0.151**	1			
6. Work engagement	0.622***	–0.244**	0.535***	–0.252**	–0.365***	1		
7. Workplace wellbeing	0.657***	–0.314**	0.580***	–0.325***	–0.405***	0.833***	1	
8. Retention intention	0.767***	–0.337**	0.602***	–0.283**	–0.420***	0.720***	0.781***	1

**p < 0.01; ***p < 0.001.

### Profiles of teaching motivation

As further evidence for identifying the best profile solution, we examined the characteristics of profiles. Table 2 displayed the fit indices of LPA. AIC, BIC, and aBIC continued to fall from the first- to fifth-profile model. Table 2 displayed the fit indices of LPA. From the first to the fifth profile model, AIC, BIC, and aBIC continued to decrease. When the fourth profile was included, the AIC, BIC, and adjusted BIC decreased, the reductions were small (△AIC = 1145.71; △BIC = 1079.56; △aBIC = 1120.85, respectively) when compared to the fit index in first three subgroups. Furthermore, the entropy of the third-profile (entropy = 0.946) was better than that of the fourth-profile (*p* = 0.004) from the VLMR and BLRT results. Notably, the fourth- and fifth-profile model produced profiles that were almost comparable to those produced by the three-profile model (based on the group disparities in these indicators). These profiles merely subdivided the percentage of specific indicators of motivation variable, with no further comparison value. Overall, the third-profile model was adopted in current study.

**TABLE 2:** Model fit results of latent profile analyses.

No. of profiles	AIC	BIC	aBIC	Entropy	VLMR	BLRT
1	38739.77	38861.91	38785.68			
2	35663.14	35851.44	35733.92	0.921	0.000	0.000
3	33753.82	34008.29	33849.47	0.946	0.000	0.000
4	32608.11	32928.73	32728.62	0.940	0.004	0.000
5	30515.53	30902.31	30660.90	0.943	0.000	0.000

Table 3 showed the average posterior probabilities of profile membership. The probabilities for the three-profile model were 97.30, 98.10, and 97.10%, respectively, indicating reasonable discriminability and reliability (Nagin, 2005).

**TABLE 3:** Average latent class probabilities for most likely class membership (row) by latent class (column).

	Profile 1	Profile 2	Profile 3	*N* (%)
1	0.973	0.027	0.000	133 (11.1%)
2	0.009	0.981	0.010	688 (57.4%)
3	0.000	0.029	0.971	378 (31.5%)

**FIGURE 2:**
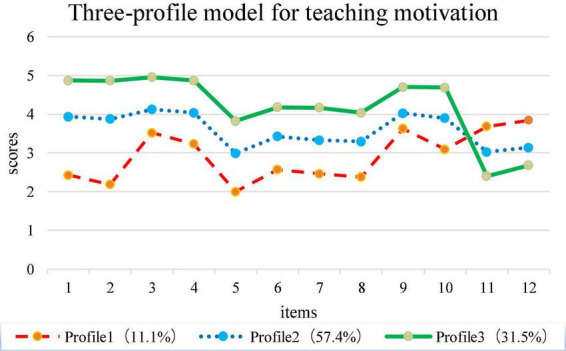
Three-profile model for kindergarten teacher motivation.

Latent means of the five motivational indicators from the third-profile were provided in Table 4. This study identified three unique motivation profiles for kindergarten teachers labeled “low value-high cost” (profile 1), “moderate all” (profile 2), and “high value-low cost” (profile 3) in Figure 2. These labels were allocated based on scores for specific indicators of motivation variables (i.e., intrinsic value, attainment value, utility value, self-efficacy, cost), and the group difference on those indicators. The “low value-high cost” (profile 1) accounted for 11.1% of the overall sample, and these kindergarten teachers in this profile showed lower intrinsic value, attainment value, utility value, moderate self-efficacy, and highest cost. For this group, Kindergarten teachers in ethnic minority areas have insufficient teaching ideal and beliefs, and the teaching profession cannot satisfy their physical needs, resulting in higher teaching pressure. Kindergarten teachers in ethnic areas with “moderate all” (profile 2) showed moderate intrinsic value, attainment value, utility value, self-efficacy and cost, accounting for 57.4% of the sample. For this group, these kindergarten teachers believed they are suitable for teaching in kindergarten to some extent, had a moderate interest in teaching and a sense of achievement, and were concerned about children’s development; they also expected the rewards of wealth and social status that come with teaching in ethnic areas, and they experienced stress and tension at work (such as facing the assessment and evaluation of the Education Bureau). Nearly 31.5% of the kindergarten teachers belonged to the “high value-low cost” profile (profile 3) and had high intrinsic value, attainment value, utility value, self-efficacy, and low cost. Kindergarten teachers in this profile experienced positive values of teaching, and they were not afraid of difficulties in teaching (such as resourcefully resolve conflicts and contradictions between parents and kindergarten).

**TABLE 4:** Adjusted standardized means and standard errors of the three motivation profiles.

Variables	Profile 1		Profile 2		Profile 3		*F*	*Post-hoc*
	*M* (95% CI)	*SE*	*M* (95% CI)	*SE*	*M* (95% CI)	*SE*		
Intrinsic value	2.29 [2.15, 2.42]^2, 3^	0.07	3.89 [3.87, 3.93]^1, 3^	0.02	0.487 [4.84, 4.89]^1, 2^	0.02	1805.36***	3 > 2 > 1
Attainment value	3.01 [2.85, 3.18]^2, 3^	0.08	4.07 [4.04, 4.09]^1, 3^	0.01	4.91 [4.89, 4.93]^1, 2^	0.01	1122.79***	3 > 1 > 2
Utility value	2.34 [2.21, 2.48]^2, 3^	0.07	3.26 [3.20, 3.31]^1, 3^	0.03	4.04 [3.95, 4.14]^1, 2^	0.05	230.41**	3 > 1 > 2
Efficacy	3.56 [3.41, 3.70]^2, 3^	0.07	3.97 [3.94, 3.99]^1, 3^	0.01	4.69 [4.65, 4.74]^1, 2^	0.02	409.58**	3 > 2 > 1
Cost	3.78 [3.63, 3.92]^2, 3^	0.07	3.08 [3.02, 3.15]^1, 3^	0.03	2.55 [2.42, 2.67]^1, 2^	0.06	79.75***	3 > 2 > 1
Engagement	2.92 [2.77, 3.07]^2, 3^	0.08	3.68 [3.64, 3.72]^1, 3^	0.02	4.47 [4.41, 4.53]^1, 2^	0.03	386.577***	3 > 2 > 1
Wellbeing	2.96 [2.79, 3.12]^2, 3^	0.08	3.85 [3.82, 3.89]^1, 3^	0.02	4.62 [4.57, 4.70]^1, 2^	0.03	446.179***	3 > 2 > 1
Retention intention	2.62 [2.47, 2.76]^2, 3^	0.07	3.76 [3.72, 3.80]^1, 3^	0.02	4.67 [4.63, 4.71]^1, 2^	0.02	839.578***	3 > 2 > 1

1, Profile 1; 2, profile 2; 3, profile 3.

### Outcomes of kindergarten teachers’ motivation profiles

**FIGURE 3:**
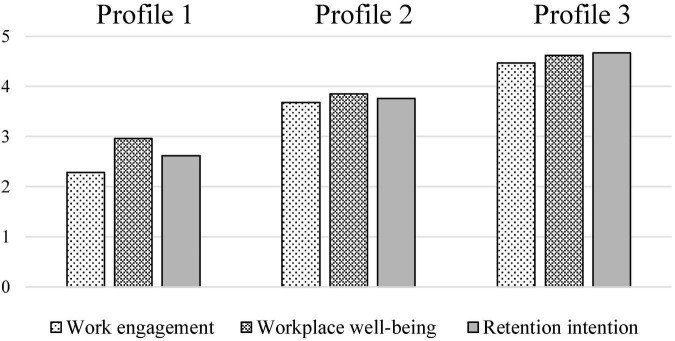
Outcome levels in the final 3 profile solution.

Table 4 displayed the means of these outcome variables, and statistical significance of the comparison at the level of each pairwise outcome variables for the three profiles. Figure 3 depicted the exact means of the three outcome variables.

The results indicated that the “low value-high cost” (profile 1) had significantly lower work engagement, workplace wellbeing, and retention intention than the “moderate all” (profile 2) and “high value-low cost” (profile 3). Among the three profiles, the “high value-low cost” (profile 3) had the most positive effects on work engagement, workplace wellbeing, and retention intention.

### Examination of the mediation model

**FIGURE 4:**
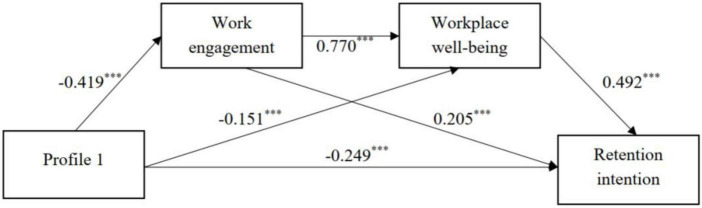
Mediating role of work engagement and workplace wellbeing in the association between “low motivation-high cost” (profile 1) and retention intention. Moderate all (profile 2) was the reference group of kindergarten teacher motivation profiles. Unstandardized path coefficients were reported.

**FIGURE 5:**
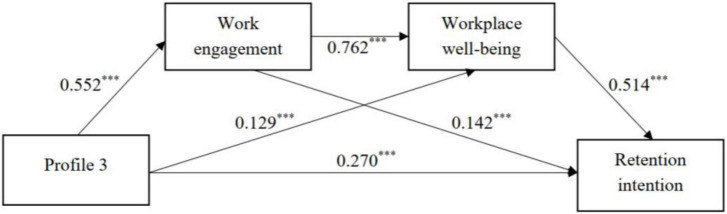
Mediating role of work engagement and workplace wellbeing in the association between “high value-low cost” (profile 3) and retention intention. Moderate all (profile 2) was the reference group of kindergarten teacher motivation profiles. Unstandardized path coefficients are reported.

Figures 4, 5 illustrated the findings of chained mediation analysis. All the models’ effects indices were reported in Tables 5, 6. With “moderate all” (profile 2) as the reference group, “low motivation-high cost” (profile 1) had direct effects (β = –0.249, *p* < 0.001) on retention intention. The “low motivation-high cost” (profile 1) was related with lower work engagement (β = –0.419, *p* < 0.001). Work engagement was significantly linked to workplace wellbeing (β = 0.770, *p* < 0.001), and workplace wellbeing was significantly related with retention intention (β = 0.492, *p* < 0.001). Further study performed the bootstrapping method. Repeated samples were taken 1,000 times and 95% confidence intervals were calculated. The results ranged from –0.510 to –0.326 (not including zero), indicating that work engagement and workplace wellbeing could partially mediate the association between “low motivation-high cost” (profile 1) and retention intention. Specifically, results revealed that the total mediating effect of the three mediating paths was –0.319. The total effect was 0.568, which was the sum of the direct and total mediation effects. The effect size was the value of each mediation effect divided by the total effect. The effect size of the three mediation paths was 15.14, 13.03, and 27.99%, respectively.

**TABLE 5:** Models’ effects indices.

Effect	Path relationship	Effect size	Bootstrapping (95% CI)	Relative mediation effect (%)
Direct effect	Profile 1 → retention intention	–0.249	[–1.191, –0.883]	43.84
Indirect effect	Profile 1 → engagement → retention intention	–0.086	[–0.314, –0.143]	15.14
	Profile 1 → wellbeing → retention intention	–0.074	[–0.288, –0.131]	13.03
	Profile 1 → engagement → wellbeing → retention intention	–0.159	[–0.510, –0.326]	27.99
Total indirect effect		–0.319	[–1.612, –1.318]	56.16
Total effect		–0.568		100

Low motivation-high cost = profile 1.

**TABLE 6:** Models’ effects indices.

Effect	Path relationship	Effect size	Bootstrapping (95% CI)	Relative mediation effect (%)
Direct effect	Profile 3 → retention intention	0.270	[0.840, 0.988]	42.86
Indirect effect	Profile 3 → engagement → retention intention	0.078	[0.060, 0.208]	12.38
	Profile 3 → wellbeing → retention intention	0.066	[0.075, 0.162]	10.47
	Profile 3 → engagement → wellbeing → retention intention	0.216	[0.306, 0.454]	34.29
Total indirect effect		0.36	[1.024, 1.152]	57.14
Total effect		0.63		100

High value-low cost = profile 3.

With “moderate all” (profile 2) as the reference group, “high value-low cost” (profile 3) had direct effects (β = 0.270, *p* < 0.001) on retention intention. The “high value-low cost” (profile 3) was associated with higher (β = 0.552, *p* < 0.001) work engagement. Work engagement was significantly connected with workplace wellbeing (β = 0.762, *p* < 0.001), and workplace wellbeing was significantly connected with retention intention (β = 0.514, *p* < 0.001). A bootstrapping method was also used to further analysis mediating effect (1,000 bootstrap). This finding revealed a range of 1.024–1.152 (not including zero), indicating that work engagement and workplace wellbeing could partially mediate the association between “low motivation-high cost” (profile 2) and retention intention. Specifically, the results revealed that the total mediating effect of the three mediating paths was 0.360. The total effect was −0.521. The effect size of the three mediation paths is 12.38, 10.47, and 34.29%, respectively.

## Discussion

This study adds to our understanding of kindergarten teachers’ motivation by (1) modeling the complexity of kindergarten teachers’ motivation using LPA; (2) evaluating the relevance of kindergarten teachers’ profiles for outcome variables, namely, work engagement, workplace wellbeing, and retention intention; and (3) exploring the association mechanism between kindergarten teacher profiles and retention intention. These findings contribute to prior study by developing a holistic view of the complicated system that links work motivation, antecedents, and outcomes among kindergarten teachers in ethnic minority areas in China.

### Kindergarten teachers’ motivation profiles

The primary goal of our research was to establish the motivation profiles of kindergarten teachers based on EVT. The findings, which were consistent with earlier study, revealed that kindergarten teachers from ethnic minority areas in China may have more than one reason for choosing teacher as their profession.

Among the work motivation of kindergarten teachers, three distinct profiles were identified: “low value-high cost,” “moderate all,” and “high value-low cost.” The most common profile was “moderate all,” which characterized by relatively moderate motivation indicators (i.e., intrinsic value, attainment value, utility value, self-efficacy, and cost). This indicated that most teachers (57.4%) had profiles that indicated patterns of moderate all in all indicators. 31.5% kindergarten teachers belonged to the “high value-low cost,” which was the most idealized configurations of teachers. Because these teachers not only had positive intrinsic value, attainment value, utility value and higher efficacy in teaching, but also had lowest cost, which meant that teachers have the confidence to actively overcome difficulties in teaching. The proportion of “low value-high cost” (11.1%) was the lowest, resulting in the lowest intrinsic value, attainment value, utility value, self-efficacy, and highest cost. This is because teachers’ teaching behavior was influenced by a low sense of achievement, material dissatisfaction, a lack of interest in teaching, low teaching efficiency and great work pressure.

These findings also supported the EVT that a teacher’s motivation to teach is the result of a conjunction of motivating indicators. Hence, a sole factor would not be sufficient to accurately predict a teacher’s motivation behavior, and numerous positive elements could be mobilized to motivate kindergarten teachers to engage in optimum teaching behavior. The study also sheds light on the association between kindergarten teachers work motivation and the geographical areas they are working in. Previous research have indicated that most kindergarten teachers regarded teaching as a demanding and stressful profession (Zhang et al., 2019; Rad et al., 2022a), especially for those who work in metropolitan areas (Cao, 2008). Nonetheless, this study found that several kindergarten teachers in ethnic minority areas demonstrated a relatively positive work motivation pattern, which could be explained by China’s affirmative action policies favoring ethnic minority areas. For instance, since 2010, the government has implemented a variety of educational reforms aimed at increasing teachers’ motivation to teach in ethnic minority areas, including meeting their material needs (e.g., decent salary), providing more opportunities for professional development and promotion, and forging a comfortable work environment (Long, 2021; Long and Yuan, 2021).

### Differences in outcome variables among profiles

The present study’s third aim was to investigate if teacher motivation profiles differ in different outcome variables (work engagement, workplace wellbeing, and retention intention). The study discovered some differences across the three motivation profiles in terms of engagement, workplace wellbeing, and retention intention. The “high value-low cost” profile reported better work engagement, workplace wellbeing, and retention intention than the other profiles. The “moderate all” profile exhibited less work engagement, workplace wellbeing and retention intention than “high value-low cost” profile. Kindergarten teachers with the “high value-low cost” profile appear to be more advantaged than those with the other profiles, because positive work attitudes of teachers will promote better work performance, which will subsequently support education quality development (Rad et al., 2022b). Kindergarten teachers with the “low value-high cost” profile, on the other hand, appear to be at risk because they showed the least work engagement, workplace wellbeing, and retention intention, which could lead to attrition and shortages of teachers (Hong et al., 2021), affecting the quality of kindergarten education in minority areas (Long, 2021). These findings supported previous researches (Li et al., 2015; Howard et al., 2016; Zhang et al., 2019), demonstrating that more favorable motivation profiles were link to higher work engagement, workplace wellbeing, and retention intention.

### Associations between kindergarten teacher motivation profiles and outcomes

This study’s fourth aim was to examine the underlying mechanism between the work motivation profiles and kindergarten teachers’ retention intention in ethnic minority areas in China. It was found that work engagement and workplace wellbeing emerged as mediators between work motivation profiles (“high value-low cost” profile; “low value-high cost” profile) and retention intention.

Regarding the first mediation link (i.e., motivation profiles-work engagement-retention intention), we found that the kindergarten teachers in the “low value-high cost” profile (“high value-low cost” profile) had a lower (higher) work engagement, compared to those in the “moderate all” profile, which was aligned with conservation of resources theory. Because processing resources (such as motivation) promotes the acquisition of extra resources (Hobfoll, 2002). Previously, similar accumulated effects on work engagement have been established (Valero and Hirschi, 2016). Furthermore, the findings also demonstrated that people who perceive higher (lower) work engagement are more (less) motivated to stay at the same organization, which in line with Sánchez-Cardona et al. (2021)’s view. According to the above views, positive motivation (i.e., “high value-low cost” profile) may initiate a behavior process that makes work more engaging, resulting in a desire to remain in the same organization.

In terms of the second mediation link (i.e., motivation profiles-workplace wellbeing-retention intention), findings indicated that kindergarten teachers in the “low value-high cost” profile (“high value-low cost” profile) had lower (higher) workplace wellbeing than those in the “moderate all” profile, which is consistent with person-organization fit theory. This meant that when employees’ motivations are highly aligned with those of their job, they are more likely to respond positively to organizational practices, resulting in increased positive affect and attitudes at work, indicating job wellbeing (Fu et al., 2020; Samfira and Paloş, 2021). Additionally, the results also confirmed that individuals who perceive higher (lower) workplace wellbeing are more (less) motivated to stay with their current post, which consistent with model of wellbeing improvement and employer outcomes (Sears et al., 2013). That is, workplace wellbeing could influence employees’ psychological disengagement from work and their capacity to perform, which may ultimately result in resignation or long–term employment (Sears et al., 2013; Bardach et al., 2022). According to the above perspective, more motivated teachers (those with a “high value-low cost” profile) experienced a greater sense of workplace wellbeing that might predict teachers’ retention intention.

Finally, the chain mediation model demonstrated that kindergarten teacher work motivation profiles could indirectly influence their retention intention via the chain-mediated effect of work engagement and workplace wellbeing. Specifically, compared with those in the “moderate all” profile, kindergarten teachers in the “low value-high cost” profile (“high value-low cost” profile) could decrease (enhance) work engagement, which may in turn affect kindergarten teachers’ workplace wellbeing and further predict their retention intention, in line with Job Demands Resources Model. This study also suggested that the mechanism of work motivation profiles influencing kindergarten teachers’ retention intention was complicated. Even though this study identified independent mediating effects of work engagement and workplace wellbeing, and the chain mediating effect they generate, all three paths had partial mediating effects. We found that, among all the paths in which kindergarten teachers’ work motivation profiles affect their retention intention, the chain mediating path had the greatest indirect effect value (“high value-low cost” profile → work engagement → workplace well-being → retention intention). This finding indicated that “high value-low cost” profile of kindergarten teachers was the most ideal group, because these kindergarten teachers had a stronger retention intention and demonstrated positive internal work experience. Therefore, among effort to retain kindergarten teachers in China’s ethnic minorities, greater emphasis should be placed on improving their enthusiasm and developing their internal psychological resources.

### Research implications

Our findings have some practical implications for understanding kindergarten teachers’ motivation profiles and the important factors associated with it. First, the study indicated that expectancy of success, attainment value, intrinsic value, utility value and cost may help differentiate motivation profiles. This provides an effective intervention method by satisfying teachers’ demands, identifying the deficiencies in the teaching profession, and assisting them in coping with challenges.

Second, these results suggest that minority kindergarten teachers with lower education level were more motivated at work, while highly educated teachers were less likely to be motivated and therefore more likely to turnover. Therefore, such a dilemma posed a challenge for education administrators and stakeholders—how to balance among teachers’ effectiveness, motivation, and retention. One strategy is to promote holistic programs that integrate kindergarten teacher motivation and desired outcomes (work engagement, workplace wellbeing, and retention intention).

### Limitations

This study has limitations that need to be addressed. First, while self-report was adequate for measuring psychological characteristics, the accuracy of the results could be further increased should more additional objective indicators and assessments be used. Second, additional predictive indicators and outcome variables for work motivation could be considered in order to improve the predictive effect of motivation profiles. For instance, minority policy, past practical experiences, or emotions may all have differing associations with these characteristics. Third, the cross-sectional methodology would not be able to provide a definitive conclusion about causation, and a longitudinal study should be used to analyze changes in kindergarten teacher profiles and to highlight the interaction of these variables. Finally, the profiles in a northwest ethnic minority context has limited implications for other cultural backgrounds, as cultural differences could lead to variation in the number and configuration of the teacher motivation profiles. Identifying profiles may differ in different provinces of China and other nations with their own education evaluation systems (i.e., the developmental prospects of preschool teachers vary by region in China).

## Conclusion

This study firstly classified kindergarten teachers’ motivation profiles in ethnic minority areas in China. The findings revealed that kindergarten teachers may have more than one reason for choosing teachers as their profession. Secondly, kindergarten teacher with different motivation profiles have significant differences in work engagement, workplace wellbeing, and retention intention. Finally, work engagement and workplace wellbeing mediated the association between motivation profiles and retention intention.

## Data availability statement

The original data in this study are available from DS (dashengshi@yahoo.com) or MZ (zhangmm817@foxmail.com) upon request.

## Ethics statement

The studies involving human participants were reviewed and approved by the Institutional Review Board (IRB) of Minzu University of China. The participants provided their written informed consent to participate in this study.

## Author contributions

DS and MZ planned the design of the study, organized the data collection and formal analysis, and drafted the original draft. MZ, YC, RJ, and XY reviewed and edited the draft. All authors contributed to the manuscript and approved the submitted version.
